# Negative oncologic impact of poor postoperative pain control in left-sided pancreatic cancer

**DOI:** 10.3748/wjg.v23.i4.676

**Published:** 2017-01-28

**Authors:** Eun-Ki Min, Jae Uk Chong, Ho Kyoung Hwang, Sang Joon Pae, Chang Moo Kang, Woo Jung Lee

**Affiliations:** Eun-Ki Min, Yonsei University College of Medicine, Seoul 03722, South Korea; Jae Uk Chong, Ho Kyoung Hwang, Chang Moo Kang, Woo Jung Lee, Department of Hepatobiliary and Pancreatic Surgery, Yonsei University College of Medicine, Seoul 03722, South Korea; Jae Uk Chong, Ho Kyoung Hwang, Chang Moo Kang, Woo Jung Lee, Pancreaticobiliary Cancer Clinic, Yonsei Cancer Center, Severance Hospital, Seoul 03722, South Korea; Sang Joon Pae, National Health Insurance Corporation Ilsan Hospital, Goyang 10444, South Korea

**Keywords:** Pancreatic cancer, Pancreatectomy, Survival, Postoperative pain, Recurrence

## Abstract

**AIM:**

To investigate the association between postoperative pain control and oncologic outcomes in resected pancreatic ductal adenocarcinoma (PDAC).

**METHODS:**

From January 2009 to December 2014, 221 patients were diagnosed with PDAC and underwent resection with curative intent. Retrospective review of the patients was performed based on electronic medical records system. One patient without records of numerical rating scale (NRS) pain intensity scores was excluded and eight patients who underwent total pancreatectomy were also excluded. NRS scores during 7 postoperative days following resection of PDAC were reviewed along with clinicopathologic characteristics. Patients were stratified into a good pain control group and a poor pain control group according to the difference in average pain intensity between the early (POD 1, 2, 3) and late (POD 5, 7) postoperative periods. Cox-proportional hazards multivariate analysis was performed to determine association between postoperative pain control and oncologic outcomes.

**RESULTS:**

A total of 212 patients were dichotomized into good pain control group (*n* = 162) and poor pain control group (*n* = 66). Median follow-up period was 17 mo. A negative impact of poor postoperative pain control on overall survival (OS) was observed in the group of patients receiving distal pancreatectomy (DP group; 42.0 mo *vs* 5.0 mo, *P* = 0.001). Poor postoperative pain control was also associated with poor disease-free survival (DFS) in the DP group (18.0 mo *vs* 8.0 mo, *P* = 0.001). Patients undergoing pancreaticoduodenectomy or pylorus-preserving pancreaticoduodenectomy (PD group) did not show associations between postoperative pain control and oncologic outcomes. Poor patients’ perceived pain control was revealed as an independent risk factor of both DFS (HR = 4.157; 95%CI: 1.938-8.915; *P* < 0.001) and OS (HR = 4.741; 95%CI: 2.214-10.153; *P* < 0.001) in resected left-sided pancreatic cancer.

**CONCLUSION:**

Adequate postoperative pain relief during the early postoperative period has important clinical implications for oncologic outcomes after resection of left-sided pancreatic cancer.

**Core tip:** This is a retrospective review to evaluate the association between postoperative pain control and oncologic outcomes in resected pancreatic ductal adenocarcinoma. In multivariate analysis, poor patients’ perceived pain control was an independent risk factor for both disease-free survival (HR = 4.157; 95%CI: 1.938-8.915; *P* < 0.001) and overall survival (HR = 4.741; 95%CI: 2.214-10.153; *P* < 0.001) in resected left-sided pancreatic cancer. Adequate postoperative pain control to reduce patients’ perceived pain during immediate postoperative period may be as important as adjuvant therapy in resected left-sided pancreatic cancer.

## INTRODUCTION

Pancreas cancer is one of the most fatal malignancies in the world and is currently the fourth leading cause of cancer death in the United States[1]. Surgical excision remains the only curative therapy for pancreatic cancer. However, the resection rate is less than 20% at the time of initial diagnosis, and the rate of recurrence is extremely high even after surgery, occurring in up to 65% to 95% of patients[2-4]. To overcome the high incidence of micrometastatic disease, margin-negative resection[5] and the use of adjuvant treatment[2,3,6] have been considered as important prognostic factors of long-term survival. Nonetheless, 5-year overall survival remains less than 25% even after receiving adjuvant chemotherapy following resection[2,3].

Recently, the importance of the perioperative period on oncologic outcome after cancer surgery has been emphasized in several review studies[7-10]. These studies underlined that the paracrine and neuroendocrine responses caused by surgical stress could promote tumor metastasis through direct action on residual malignant cells and by suppressing natural killer (NK) cell activity, thus compromising antimetastatic cell-mediated immunity (CMI)[8,11,12]. Downregulation of immunity after surgery is known to peak at postoperative day (POD) 3[13], and the decline in NK cell cytotoxicity has been documented to last until POD 7 to 9, depending on the surgical procedure[14-16]. A decrease in NK cell cytotoxicity following pancreaticoduodenectomy (PD) at POD 7 was also recently reported[17]. These results indicate that the early postoperative period harbors potential for the initiation of cancer metastasis, either *de novo* or from pre-existing micrometastasis.

Even when surgeons achieve R0 resection, various factors of this disproportionally pivotal perioperative period can facilitate growth of potential residual cancer beyond a critical immunological threshold, leading to cancer recurrence. Suggested perioperative risk factors that modulate surgery-induced immunosuppression include anesthetic technique, analgesic agents, blood transfusion, hypothermia, and pain[7-10].

Among these factors, acute pain is known to suppress NK cell activity[18], and its immunosuppressive properties have been shown to promote tumor growth in animal models[19-22]. Postsurgical pain activates the sympathetic nervous system (SNS), leading to catecholamine secretion[23], which directly inhibits NK cells. Furthermore, postoperative pain is not only a result of surgical tissue damage and nociception, but also reflects psychological stress, which has been reported as a risk factor of metastatic progression in some clinical trials[24,25].

In spite of its potential role as an immunomodulator promoting tumor growth and metastasis, there has been no study to evaluate the oncologic significance of postoperative pain following resection of pancreas cancer. In this study, we investigated the association between postoperative pain control and oncologic outcomes in resected pancreatic ductal adenocarcinoma (PDAC).

## MATERIALS AND METHODS

### Patients and study design

**Figure 1:**
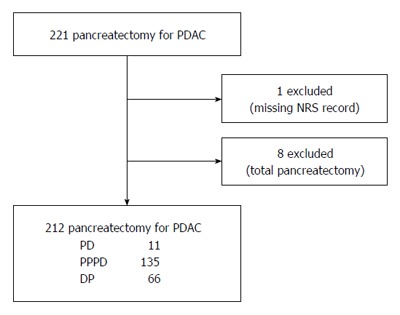
Patient eligibility. PDAC: Pancreatic ductal adenocarcinoma; NRS: Numerical rating scale; PD: Pancreaticoduodenectomy; PPPD: Pylorus-preserving pancreaticoduodenectomy; DP: Distal pancreatectomy with or without spleen preservation.

From January 2009 to December 2014, 221 patients with PDAC underwent pancreatectomy with curative intent in our center. We retrospectively reviewed clinicopathologic characteristics and numerical rating scale (NRS) pain intensity score recorded in the nursing records system. One patient was excluded because of missing NRS data for an unknown reason and eight patients who required total pancreatectomy were also excluded (Figure 1). The study was reviewed and approved by the Institutional Review Board of Yonsei University College of Medicine.

### Data collection and analysis

NRS score from the nursing records system was available from 2009. Nurses administered the 11-point NRS, with the score ranging from 0 to 10, to evaluate pain intensity whenever the patients reported pain. Patients were instructed to rate 0 as “no pain at all”and 10 as “the worst possible pain”. NRS scores during 7 postoperative days following resection of PDAC were reviewed.

We defined early pain score as the average of all pain scores reported on POD 1, 2, and 3 and late pain score as the average of scores reported on POD 5 and 7. In consideration of the subjective nature of pain and the importance of “perceived control”, we applied the concept of pain control expressed as difference in pain intensity between the two periods rather than objective pain intensity value. We defined the “good pain control group” as the group of patients whose late pain intensity was lower than that of early pain intensity and the “poor pain control group” as the group of patients whose late pain intensity was the same or higher than the early pain intensity.

Postoperative complications were defined using the Clavien-Dindo classification of surgical complications[26]. Major complications were defined as complications with a Clavien-Dindo score of III or higher, which require additional interventional and/or medical treatment associated with prolonged hospital stay. TNM stages were classified according to the American Joint Committee on Cancer (AJCC; 7^th^ edition) staging system[27]. Multivisceral resection was defined as resection of any organ or a part of an organ other than the pancreas and spleen. Combined resection was defined as any multivisceral resection or vascular resection.

### Statistical analysis

Statistical analyses were performed using SPSS 20.0 for Windows (SPSS Inc., Chicago, IL, United States) and MedCalc 16.8.4 for Windows (MedCalc Inc., Mariakerke, Belgium). For continuous variables, *t*-test was performed and reported as mean and standard deviation. For matched data analysis, paired *t*-test was performed. Categorical variables were compared using the Chi-Square test or Fisher’s exact test and reported as number (*n*) and percentage (%). Overall survival (OS) rates and disease-free survival (DFS) rates were estimated using the Kaplan-Meier method. Log-rank test was performed to compare the categorical groups in univariate analysis. A multivariable Cox proportional hazards regression model was used to determine independent risk factors associated with OS and DFS. This model included all of the categorized patient, resection, and tumor characteristics with log-rank *P* values ≤ 0.150. Exponential (β) measures were reported with 95%CI to evaluate the risks associated with each factor. Statistical significance was achieved at *P* < 0.05.

## RESULTS

### Patients characteristics

A total of 212 patients who underwent pancreatectomy for PDAC were retrospectively reviewed. The clinicopathological characteristics are summarized in Table 1. Median follow-up period was 17 mo. Sixty-six patients (31.3%) received neoadjuvant concurrent chemoradiotherapy (CCRT) before pancreas resection, and 154 patients (72.6%) received adjuvant treatment of chemotherapy, radiotherapy, or CCRT according to their general condition. R0 resection was achieved in 187 patients (88.2%). In terms of resection methods, 146 patients (68.9%) underwent pancreaticoduodenectomy (PD) or pylorus-preserving pancreatoduodenectomy (PPPD), and 66 patients (31.1%) underwent distal pancreatectomy (DP) with or without spleen preservation.

**Table 1:** Clinicopathologic characteristics of the patients *n* (%)

**Characteristic (*n* = 212)**	**Frequency, mean ± SD**
Age (yr)	62.8 ± 9.5
Male gender	125 (59.0)
BMI (kg/m^2^)	22.8 ± 2.9
Diabetes	73 (34.4)
ASA	
1	66 (31.1)
2	92 (43.4)
3	49 (23.1)
4	2 (0.9)
Operative time (min)	402.2 ± 129.3
Intraoperative bleeding (mL)	626.0 ± 482.8
Intraoperative transfusion	47 (22.2)
pT stage	
T0	9 (4.2)
T1	16 (7.5)
T2	3 (1.4)
T3	182 (85.8)
T4	2 (0.9)
pN stage	
N0	106 (50.0)
N1	106 (50.0)
pTNM staging	
I	18 (8.5)
II	182 (85.8)
III	2 (0.9)
IV	1 (0.5)
R status	
R0	187 (88.2)
R1	23 (10.8)
R2	2 (0.9)
Cell differentiation	
Well	24 (11.3)
Moderate	152 (71.7)
Poor	17 (8.0)
Undifferentiated	1 (0.5)
Retrieved lymph nodes	17.3 ± 9.9
Vascular resection	55 (25.9)
Mutivisceral resection	36 (17.5)
Combined resection	71 (33.5)
Lymphovascular invasion	71 (33.5)
Perineural invasion	146 (68.9)
Neoadjuvant CCRT	66 (31.3)
Adjuvant treatment	154 (72.6)
Complications	
Minor	112 (52.8)
Major(≥ G3)	20 (9.4)
Length of hospital stay (d)	21.1 ± 15.0
Recurrence	137 (64.6)

ASA: American Society of Anesthesiologists; CCRT: Concurrent chemoradiotherapy.

### Changes in postoperative pain intensity following pancreatectomy

**Figure 2:**
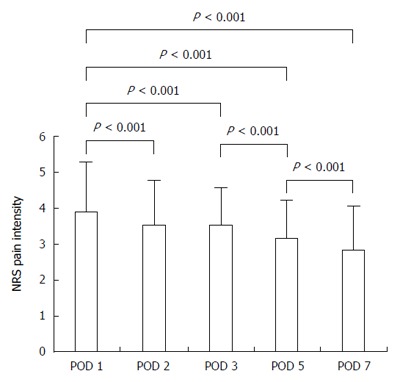
Overall changes in postoperative numerical rating scale pain intensity following pancreatectomy. POD: Postoperative day; NRS: Numerical rating scale.

For the overall patient population, postoperative pain intensity decreased significantly at POD 2, 3, 5, and 7 compared with POD 1 (*P* < 0.001 for each; Figure 2). There was a significant decrease in pain intensity between each two successive days (*P* < 0.001), except between POD 2 and 3 (*P* = 0.916).

**Figure 3:**
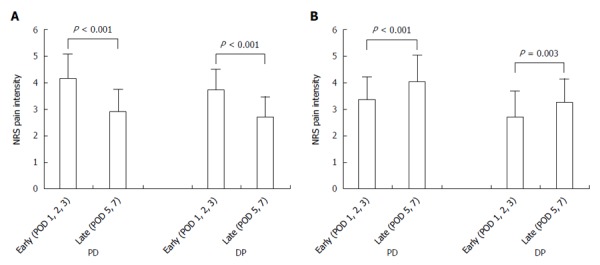
Change in numerical rating scale pain intensity following pancreatectomy stratified by quality of pain control. A: Good pain control group (*n* = 109, PD; *n* = 53, DP); B: Poor pain control group (*n* = 37, PD; *n* = 13, DP). PD: Patients underwent pancreaticoduodenectomy or pylorus-preserving pancreaticoduodenectomy; DP: Patients underwent distal pancreatectomy; POD: Postoperative day; NRS: Numerical rating scale.

Patients were divided into a good pain control group (*n* = 162) and poor pain control group (*n* = 50). The good pain control group showed a reduction of pain intensity from 4.13 ± 0.93 to 2.87 ± 0.86 for the PD group (*n* = 109, *P* < 0.001) and from 3.71 ± 0.77 to 2.69 ± 0.78 for the DP group (*n* = 53, *P* < 0.001, Figure 3A). The poor pain control group showed an increase of pain intensity from 3.35 ± 0.87 to 4.03 ± 1.02 for the PD group (*n* = 37, *P* < 0.001) and from 2.71 ± 0.99 to 3.26 ± 0.88 for the DP group (*n* = 13, *P* = 0.003, Figure 3B).

### Oncologic impact of postoperative pain intensity following pancreatectomy

**Figure 4:**
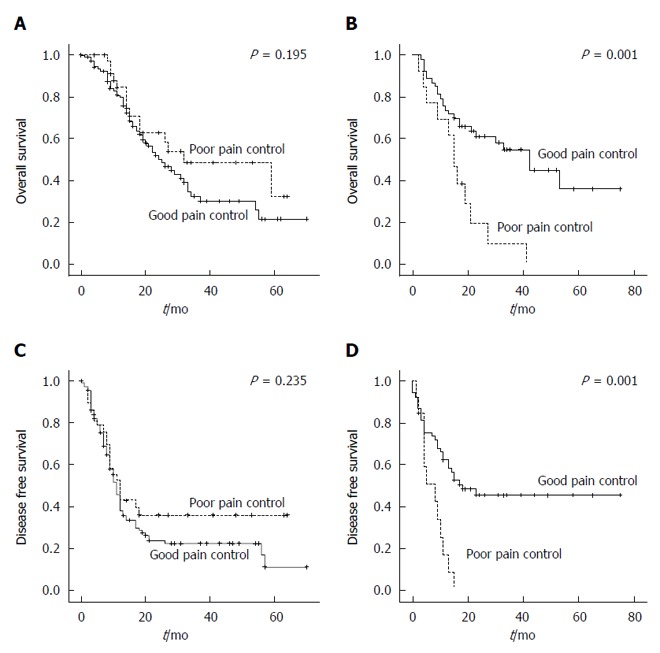
Oncologic outcomes. Comparison of overall survival between the good pain control group (solid line) and poor pain control group (dotted curve) after pancreaticoduodenectomy (PD) (A) and distal pancreatectomy (DP) (B); Comparison of disease-free survival between the good pain control group (solid line) and poor pain control group (dotted curve) after PD (C) and DP (D).

A negative impact of poor postoperative pain control on OS was observed in the DP group [good pain control *vs* poor pain control, median survival 42.0 mo (95%CI: 26.2-57.8) *vs* 15.0 mo (95%CI: 11.5-18.5), *P* = 0.001, Figure 4B]. Also, poor pain control exerted a negative effect on DFS in the DP group (good pain control *vs* poor pain control, median 18.0 mo *vs* 8.0 mo, *P* = 0.001, Figure 4D). Patients undergoing pancreaticoduodenectomy or pylorus-preserving pancreaticoduodenectomy (PD group) did not show associations between postoperative pain control and oncologic outcomes.

### Comparison between the good pain control group and the poor pain control group among patients undergoing distal pancreatectomy

There were no significant differences in preoperative, intraoperative, or postoperative outcomes between the good pain control group and the poor pain control group (*P* > 0.05, Table 2). Other interesting finding was that surgical approach, such as open or minimally invasive, did not influence categorization of pain response (*P* = 0.523). Whether the patients received neoadjuvant CCRT or not also did not affect grouping of pain control (*P* = 0.719). There were also no differences in method of postoperative pain management techniques between the group (*P* = 0.445).

**Table 2:** Clinicopathologic differences between the good pain control group and poor pain control group undergoing distal pancreatectomy *n* (%)

	**Pain control group**		***P* value**
	**Good** **(*n* = 53)**	**Poor** **(*n* = 13)**	
Age (yr)	65.6 ± 8.2	63.2 ± 11.3	0.378
Male gender	30 (56.6)	7 (53.8)	0.858
BMI (kg/m^2^)	23.1 ± 2.7	23.1 ± 2.4	0.993
Diabetes	17 (32.1)	3 (23.1)	0.739
ASA			
1	16 (30.2)	5 (41.7)	0.342
2	22 (41.5)	6 (50.0)	
3	15 (28.3)	1 (8.3)	
Surgical approach			
Open	35 (66.0)	7 (53.8)	0.523
MIS	18 (34.0%)	6 (46.2)	
Spleen preservation	7 (13.2)	2 (15.4)	> 0.999
Operation time (min)	254.1 ± 95.3	304.6 ± 89.0	0.088
Intraoperative bleeding (mL)	364.2 ± 296.0	470.38 ± 624.8	0.373
Intraoperative transfusion	5 (9.4)	3 (23.1)	0.185
Tumor size (cm)			0.407
< 3	30 (56.6)	9 (69.2)	
≥ 3	23 (43.4)	4 (30.8)	
pT stage			0.148
T0	3 (5.7)	0 (0)	
T1	3 (5.7)	2 (15.4)	
T2	2 (3.8)	1 (7.7)	
T3	45 (84.9)	9 (69.2)	
T4	0 (0.0)	1 (7.7)	
pN stage			0.851
N0	27 (50.9)	7 (53.8)	
N1	26 (49.1)	6 (46.2)	
pTNM staging			0.429
I	5 (9.5)	2 (15.4)	
II	44 (83.0)	10 (77.0)	
III	0 (0.0)	1 (7.7)	
IV	1 (1.9)	0 (0.0)	
R status			0.121
R0	47 (88.7)	11 (84.6)	
R1	6 (11.3 )	1 (7.7)	
R2	0 (0.0 )	1 (7.7)	
Retrieved lymph nodes	14.8 ± 11.0	14.2 ± 6.6	0.735
Multivisceral resection	12 (22.6)	2 (15.4)	0.718
Combined resection	14 (26.4)	2 (15.4)	0.496
Lymphovascular invasion	14 (27.5)	4 (33.3)	0.729
Perineural invasion	34 (66.7)	5 (41.7)	0.185
Grade			0.499
Well	3 (6.4)	2 (16.7)	
Moderate	38 (80.9)	9 (75.0)	
Poor	6 (12.8)	1 (8.3)	
Neoadjuvant CCRT	12 (22.6)	4 (30.8)	0.719
Preoperative CA19-9			0.154
< 300	34 (69.4)	12 (92.3)	
≥ 300	15 (30.6)	1 (7.7)	
Adjuvant treatment	39 (73.6)	9 (69.2)	0.739
Time to adjuvant treatment (d)	53.7 ± 36.8	63.0 ± 47.0	0.520
Complications			
Minor	33 (62.3)	7 (53.8)	0.578
Major(≥ G3)	3 (7.3)	2 (28.6)	0.148
Use of PCA			0.445
IV PCA	34 (64.2)	10 (76.9)	
Epidural PCA	17 (32.1)	2 (15.4)	
None	2 (3.8)	1 (7.7)	
Length of hospital stay (d)	17.1 ± 11.0	27.2 ± 45.9	0.446

ASA: American Society of Anesthesiologists; CCRT: Concurrent chemoradiotherapy; MIS: Minimal invasive surgery; PCA: Patient-controlled analgesia.

### Independent prognostic factors in resected left-sided pancreatic cancer

In univariate analysis, intraoperative transfusion, positive lymph node status, greater tumor diameter (≥ 3 cm), and poor pain control were identified as prognostic factors for predicting DFS in resected left-sided pancreatic cancer (*P* = 0.005, *P* = 0.011, *P* = 0.028, *P* = 0.001, respectively; Table 3). For OS, longer operation time (≥ 300 min), positive lymph node status, greater tumor diameter (≥ 3 cm), multivisceral resection, not receiving adjuvant treatment, and poor pain control were significant prognostic factors in univariate analysis (*P* = 0.035, *P* = 0.020, *P* = 0.023, *P* = 0.043, *P* = 0.017, *P* = 0.001, respectively; Table 3). Subsequent multivariate analysis revealed positive lymph node status, greater tumor diameter (≥ 3 cm), not receiving adjuvant treatment, and poor pain control as independent risk factors for both DFS and OS in resected left-sided pancreatic cancer (Table 4).

**Table 3:** Univariate analysis of factors affecting disease-free survival and overall survival after distal pancreatectomy

	***n***	**DFS**		**OS**	
		**Median survival (mo)**	***P* value^1^**	**Median survival (mo)**	***P* value^1^**
Age					
< 65	30	15	0.216	42	0.150
≥ 65	36	11		23	
Sex					
Female	29	11	0.136	27	0.829
Male	37	18		33	
BMI (kg/m^2^)					
< 25	52	11	0.209	30	0.384
≥ 25	14			27	
Diabetes					
No	46	13	0.674	27	0.654
Yes	20	15			
ASA					
1/2	49	13	0.569	30	0.903
3/4	16	15			
Surgical approach					
Open	42	14	0.971	30	0.645
MIS	24	11		41	
Spleen preservation					
Yes	9	15	0.931	27	0.619
No	57	13		33	
Operation time (min)					
< 300	39	17	0.254	41	0.035
≥ 300	27	13		15	
Bleeding (mL)					
< 500	42	11	0.632	30	0.881
≥ 500	23	15		33	
Intraoperative transfusion					
No	58	15	0.005	33	0.056
Yes	8	4		13	
Resection status					
R0	58	13	0.689	30	0.382
R1/R2	8	13		21	
Lymph node status					
N0	34		0.011	41	0.020
N1	32	9		17	
Tumor size (cm)					
< 3	39	18	0.028	42	0.023
≥ 3	27	8		15	
pT stage					
≤ 2	11		0.293	21	0.949
≥ 3	55	13		33	
Multivisceral resection					
No	52	15	0.111	33	0.043
Yes	14	10		13	
Combined resection					
No	50	15	0.474	33	0.303
Yes	16	10		15	
Lymphovascular invasion					
No	45	11	0.259	23	0.762
Yes	18	18		30	
Perineural invasion					
No	24	11	0.947	21	0.621
Yes	39	15		27	
Neoadjuvant CCRT					
Yes	16	15	0.351	33	0.433
No	50	11		21	
Preop CA19-9 (U/mL)					
< 300	46	15	0.782	27	0.540
≥ 300	16	13		42	
Adjuvant treatment					
Yes	48	15	0.094	41	0.017
No	18	4		8	
Major complications (≥ G3)					
No	43	15	0.813	42	0.916
Yes	5	4		21	
Use of PCA					
Epidural	19	13	0.757	27	0.943
IV	44	15		33	
Pain control					
Good	53	18	0.001	42	0.001
Poor	13	8		15	

1 *P* values were obtained using a log-rank test. DFS: Disease-free survival; OS: Overall survival; ASA: American Society of Anesthesiologists; MIS: Minimal invasive surgery; CCRT: Concurrent chemoradiotherapy; PCA: Patient-controlled analgesia.

**Table 4:** Univariate and multivariate Cox regression analysis of factors affecting disease-free survival and overall survival after distal pancreatectomy

**Variables**	**Disease-free survival**						**Overall survival**					
	**Univariate analysis**			**Multivariate analysis**			**Univariate analysis**			**Multivariate analysis**		
	**Exp (**β**)**	**95%CI**	***P* value**	**Exp (**β**)**	**95%CI**	***P* value**	**Exp (**β**)**	**95%CI**	***P* value**	**Exp (**β**)**	**95%CI**	***P* value**
Positive lymph node status	2.183	1.162-4.101	0.011	2.259	1.150-4.437	0.018	2.105	1.094-4.053	0.02	2.501	1.218-5.134	0.012
Tumor size (≥ 3 cm)	1.943	0.999-3.781	0.028	2.215	1.130-4.341	0.021	2.030	1.016-4.055	0.023	2.662	1.282-5.529	0.009
No adjuvant treatment	1.742	0.800-3.794	0.094	2.468	1.196-5.093	0.015	2.205	0.981-4.955	0.017	4.649	2.124-10.172	< 0.001
Poor pain control	2.934	1.158-7.430	0.001	4.157	1.938-8.915	< 0.001	2.915	1.156-7.350	0.001	4.741	2.214-10.153	< 0.001
Age (≥ 65 yr)	ND			ND			1.608	0.844-3.064	0.150	1.706	0.799-3.640	0.167
Sex (Male)	0.632	0.338-1.181	0.136	0.614	0.318-1.186	0.146	ND			ND		
Operation time (≥ 300 min)	ND			ND			1.949	0.981-3.873	0.035	1.890	0.923-3.868	0.082
Intraoperative transfusion	2.788	0.903-8.612	0.005	1.745	0.688-4.425	0.241	2.159	0.729-6.396	0.056	1.986	0.750-5.257	0.167
Multivisceral resection	1.720	0.769-3.849	0.111	1.166	0.532-2.557	0.701	2.046	0.837-5.006	0.043	1.273	0.563-2.876	0.562

ND: Not determined due to lack of significance.

## DISCUSSION

Evidence showing the association of pain relief and reduced surgery-induced tumor growth was first documented by Yeager et al[28] in colon carcinoma. Since then, the protective effect of various analgesics on surgery-induced metastasis has been reported by many studies[19,20,29-31]. However, there has been no study to evaluate the oncologic significance of postoperative pain control following resection of pancreatic cancer. To the best of our knowledge, the current retrospective study is the first to suggest that early postoperative pain control can influence patient survival after DP for PDAC, regardless of the biology of the tumor, surgical approach, or treatment modality.

A possible mechanism explaining the association of poor pain control and negative oncologic outcome could include interaction of inflammation, pain, and suppressed NK cell activity in the early postsurgical period, resulting in immunosuppression, which is known to peak on POD 3. The immunosuppressive property of pain is attributed to the direct inhibition of NK cell cytotoxicity by catecholamine secreted upon SNS activation[11,12]. Also, postoperative pain is associated with increased secretion of proinflammatory cytokines such as interleukin (IL)-1β, IL-6, and tumor necrosis factor (TNF)-α. Sommer et al[32] indicated that pain and proinflammatory cytokines interact reciprocally. Pain affects the production and secretion of cytokines, and those cytokines reduce the activation threshold of peripheral nociceptors, resulting in pain augmentation. Several studies[33-35] have shown the association of pain relief in the immediate postoperative period and attenuated production of proinflammatory cytokines. The correlation between changes in proinflammatory cytokine levels and decreased NK cell response was also reported by Baxevanis et al[15]. Considering these findings, unrelieved or elevated pain intensity at the late postsurgical period (POD 5, 7) might reflect prolonged inflammation and a state of immunosuppression and thus a higher chance of tumor metastasis and recurrence, explaining the negative oncologic outcome.

Poor pain control is basically attributed to a failure of appropriate and adequate postoperative analgesic care. However, it has also been suggested that poor pain response can be a result of the patient-specific immune state before surgery[36], a sign of ongoing or forthcoming complications[37,38], or a contributory effect of perioperative psychological factors[39,40]. Since there were no significant differences in any clinicopathologic factors between the good pain control group and poor pain control group undergoing DP (Table 2), we can only speculate that inadequate postoperative pain control was the major reason for poor postoperative pain control in left-sided pancreatic cancer. Further study should include establishment of appropriate pain control protocol to minimize influence of inadequate pain control and evaluate whether there are other possible reasons for poor postoperative pain control in left-sided pancreatic cancer.

Currently, it is routine for patients undergoing pancreas cancer surgery to receive adjuvant treatment irrespective of whether R0 resection is achieved. Therefore, the postsurgical period has been viewed as a time for managing complications and improving the general condition of the patient in order to meet the physiological requirements for receiving adjuvant treatment. During this approximately two-months period, patients do not receive anticancer treatment or intervention. However, this period - especially the immediate early period - harbors a therapeutic window of anticancer treatment, as surgery-induced immunosuppression is still in its recovery phase, and the tumor burden could start to increase again. Although, present study is based on a small sample size and retrospective observation, our data suggest that pain management after DP could be more than a matter of patient recovery to receive adjuvant treatment at the appropriate time. Rather, adequate and appropriate pain control during the early postoperative period might exert a direct curative effect on left-sided pancreatic cancer.

Interestingly, the negative oncologic impact of postoperative pain control was not observed following PD. This may have been influenced by wider surgical extent involved with PD and the impact of postoperative pain control during the postoperative 7 days may be rendered ineffective. The operation time, intraoperative blood loss, and rate of intraoperative transfusion were all higher after PD compared to DP [448.3 min *vs* 264.1 min, *P* < 0.001; 717.9 cc *vs* 385.5 cc, *P* < 0.001; 39 (26.9%) *vs* 8 (12.1%), *P* = 0.017, respectively, data not shown], reflecting greater surgical stress. Increased surgical extent has been shown to be associated with higher rates of tumor metastasis[41] and delayed recovery of NK cell cytotoxicity[15]. Also, intraoperative transfusion has been repeatedly reported to modulate the postoperative immune response[42,43]. These factors may overcome the potential immune modulation and oncologic effect of pain control during the period of assessment.

Our study has several limitations. It is a retrospective study, and the number of patients in the poor pain control group after DP was small, making it difficult to reach sound conclusions. We grouped patients into good and poor pain control groups according to differences in NRS pain intensity between early (POD 1, 2, 3) and late (POD 5, 7) periods, because inflammation and immunosuppression peak on around POD 3. However, this time frame might not fit all cases and may vary according to surgical extent or approach. Future studies are needed to test various analytic approaches targeting the critical time point when postoperative pain most significantly mediates immunomodulation.

In addition, our definition of pain control groups may not fully represent pain control state. There have been reports of using satisfaction score along with pain score in fully assessing adequate pain control[44,45]. In determining pain control state, we were limited to the use of NRS pain intensity. Further studies with assessment of satisfaction score and refined definitions for pain control groups should be undertaken.

Lastly, for pain control, patients received intravenous patient-controlled analgesia (PCA) or epidural PCA (both based on fentanyl) or opiates on demand. However, disconnect timing of PCA, as well as the type and amount of analgesics used after clamping, were not investigated in this study. Opioids are believed to exert an immunosuppressive effect when they are used in the absence of pain[22]. Also, certain types of opioids, such as tramadol (but not all opioids) can overcome the immunosuppressive effects of pain, reversing the capacity of surgical stress to suppress NK cell cytotoxicity and promote tumor metastasis in animal models[20,46]. The complex interaction of pain, opioids, non-opioid analgesics, and their net effect on immunosuppression, which might have impacted oncologic outcome, was not assessed in this study. However, relationship between pain control method and postoperative pain control should be investigated further with a well-designed pain control protocol.

This study suggests that a change in patients’ perceived pain intensity in the postoperative period could influence survival outcome in resected left-sided pancreatic cancer. Unlike other prognostic factors, such as tumor size, lymph node metastasis, differentiation, lymphovascular invasion, and perineural invasion, postoperative pain is a controllable factor. Surgeons play a leading role in controlling pain during the postoperative period. In spite of compelling evidence supporting the immunologic and oncologic importance of the perioperative period, its application to the clinical field is still in its infancy. More research on underutilized modulators of this period - not only postoperative pain, but also intraoperative hypothermia, transfusion, nutritional support, and psychological intervention - is needed for the development of patient-oriented perioperative therapy against pancreatic cancer.

In conclusion, adequate postoperative pain relief during the early postoperative period has important clinical implications for oncologic outcomes after resection of left-sided pancreatic cancer.

## COMMENTS

### Background

Acute pain is known to suppress natural killer (NK) cell cytotoxicity and promote tumor growth and metastasis in preclinical models. Therefore, postoperative pain has been suggested as one of the risk factors for cancer metastasis and recurrence after oncologic surgery. Surgery-induced immunosuppression and inflammation are known to peak around postoperative day (POD) 3, and suppression of NK cell cytotoxicity lasts from approximately POD 7 to 9. The clinical significance of postoperative pain control during this critical period in patients undergoing pancreatectomy for pancreatic ductal adenocarcinoma (PDAC) has yet to be investigated.

### Research frontiers

This study contributes in discovering associations between postoperative pain control and oncologic outcomes in resected pancreatic cancer.

### Innovations and breakthroughs

In this study, patients undergoing distal pancreatectomy for left-sided pancreatic cancer were found to have poor oncologic outcomes under poor postoperative pain control. Adequate postoperative pain relief during the early postoperative period has important clinical implications for oncologic outcomes after resection of left-sided pancreatic cancer.

### Applications

Surgeons play a leading role in controlling pain during the postoperative period. Patients’ perceived postoperative pain should be actively relieved after distal pancreatectomy for pancreatic cancer to improve oncologic outcomes.

### Terminology

PD group: Patients underwent pancreaticoduodenectomy or pylorus-preserving pancreaticoduodenectomy. DP group: Patients underwent distal pancreatectomy. Good pain control group: Patients whose late pain intensity was lower than that of early pain intensity. Poor pain control group: Patients whose late pain intensity was the same or higher than the early pain intensity. Early pain intensity: Mean of all pain scores reported on POD 1, 2, and 3. Late pain intensity: Mean of pain scores reported on POD 5 and 7. Pain scores: Measurement of numerical rating scale (NRS) pain intensity score. 11-point NRS with the score ranging from 0 to 10 was used to evaluate pain intensity whenever the patients reported pain. Patients were instructed to rate 0 as “no pain at all” and 10 as “the worst possible pain”.

### Peer-review

The study evaluated the association between postoperative pain control and oncologic outcomes in resected PDAC. The results showed that poor pain control was an independent risk factor for both DFS and OS in resected left-sided pancreatic cancer, but not in patients received PD. This is very interesting.
